# Zeolite and Neurodegenerative Diseases

**DOI:** 10.3390/molecules29112614

**Published:** 2024-06-02

**Authors:** Stefan Panaiotov, Lyubka Tancheva, Reni Kalfin, Polina Petkova-Kirova

**Affiliations:** 1 National Centre of Infectious and Parasitic Diseases, Yanko Sakazov Blvd. 26, 1504 Sofia, Bulgaria; spanaiotov@yahoo.com; 2 Institute of Neurobiology, Bulgarian Academy of Sciences, Acad. G. Bonchev Str. 23, 1113 Sofia, Bulgaria; l.tancheva@inb.bas.bg; 3 Department of Healthcare, Faculty of Public Health, Healthcare and Sport, South-West University, 66 Ivan Mihailov St., 2700 Blagoevgrad, Bulgaria

**Keywords:** zeolite, clinoptilolite, Alzheimer’s disease, Parkinson’s disease, detoxifying, anti-inflammatory, antioxidative effect

## Abstract

Neurodegenerative diseases (NDs), characterized by progressive degeneration and death of neurons, are strongly related to aging, and the number of people with NDs will continue to rise. Alzheimer’s disease (AD) and Parkinson’s disease (PD) are the most common NDs, and the current treatments offer no cure. A growing body of research shows that AD and especially PD are intricately related to intestinal health and the gut microbiome and that both diseases can spread retrogradely from the gut to the brain. Zeolites are a large family of minerals built by [SiO_4_]^4−^ and [AlO_4_]^5−^ tetrahedrons joined by shared oxygen atoms and forming a three-dimensional microporous structure holding water molecules and ions. The most widespread and used zeolite is clinoptilolite, and additionally, mechanically activated clinoptilolites offer further improved beneficial effects. The current review describes and discusses the numerous positive effects of clinoptilolite and its forms on gut health and the gut microbiome, as well as their detoxifying, antioxidative, immunostimulatory, and anti-inflammatory effects, relevant to the treatment of NDs and especially AD and PD. The direct effects of clinoptilolite and its activated forms on AD pathology in vitro and in vivo are also reviewed, as well as the use of zeolites as biosensors and delivery systems related to PD.

## 1. Neurodegenerative Diseases

“Neurodegenerative diseases” is a term used to collectively describe a group of pathological conditions manifested by different symptoms but all characterized by progressive degeneration and death of neurons and eventually general organism disability.

Neurodegenerative diseases can be classified according to the anatomic distribution of the neurodegeneration (e.g., frontotemporal degenerations, extrapyramidal disorders, or spinocerebellar degenerations) or the principal molecular abnormality (e.g., amyloidoses, tauopathies, α-synucleinopathies, and transactive response DNA binding protein of 43 kDa (TDP-43) proteinopathies) or, most commonly, according to their clinical presentations [1]. According to the generally observed clinical symptoms, neurodegenerative diseases are dementia-type diseases such as Alzheimer’s disease, frontotemporal dementia, chronic traumatic encephalopathy (CTE), Lewy body dementia and limbic predominant age-related TDP-43 encephalopathy (LATE) characterized by confusion, memory loss, cognitive impairments, and behavioral changes; demyelinating diseases with myelin damage or loss, with examples like multiple sclerosis and neuromyelitis optica spectrum disorder (NMOSD) characterized by numbness, muscle spasms, coordination issues and fatigue, weakness, and paralysis; parkinsonism-type diseases such as Parkinson’s disease and other forms of parkinsonism characterized by slowed movements, balance problems, shaking and tremors, scuffling steps, and hunched posture; motor neuron diseases with examples like amyotrophic lateral sclerosis (ALS, known as Lou Gehrig’s disease as well) and progressive supranuclear palsy (PSP) with loss of muscle control, weakness, and eventually paralysis; and prion diseases such as Creutzfeldt–Jakob disease [2].

Neurodegenerative diseases affect millions of people worldwide, and most of them, including Alzheimer’s disease and Parkinson’s disease, are strongly related to aging. One in ten individuals at the age of 65 and above has AD, and its prevalence continues to increase with increasing age [3]. At the same time, according to the World Health Organization, by 2030, the share of the population aged 60 years and over will increase from 1 billion in 2020 to 1.4 billion. By 2050, the world’s population of people aged 60 years and older will double (2.1 billion), and the number of people aged 80 years or older is expected to triple between 2020 and 2050 to reach 426 million [4].

Regardless of the rising number of people suffering from neurodegenerative diseases and the immense social burden it is imposing on society, current treatments help relieve many of the symptoms associated with neurodegenerative diseases but do not slow their progression and offer no cure.

While extensive research is going on to find the missing effective cure, it is known that a combination of genetic and environmental factors contributes to the risk of developing a neurodegenerative disease. Among the environmental factors are pesticides, fungicides, and insecticides; chemicals used in industry or consumer products (e.g., polychlorinated biphenyls); air, water, and soil pollution; metals (e.g., lead, cadmium, manganese, copper, and aluminium); and biological factors (e.g., endotoxins produced by bacteria) and dietary and lifestyle factors (e.g., smoking and unhealthy diet). Moreover, although neurodegenerative diseases are typically defined by specific protein aggregates (assemblies) (such as amyloid-β (Aβ) plaques and hyperphosphorylated tau protein aggregates as in AD, TDP-43 as in LATE and ALS, and α-synuclein as in PD and Lewy body disorders), they share many common fundamental features linked to progressive neuronal dysfunction and death such as oxidative stress, programmed cell death, and neuroinflammation [1,5,6]. Thus, targeting those proccesses, especially simultaneously, could ensure effective therapy against more than one neurodegenerative condition.

## 2. Gut Health and Alzheimer’s Disease and Parkinson’s Disease

The most common neurodegenerative diseases are Alzheimer’s disease and Parkinson’s disease.

Alzheimer’s disease (AD) is characterized by the deposition of intracellular neurofibrillary tangles (NFTs), composed of a highly phosphorylated form of the tau protein, and extracellular senile plaques, composed of insoluble aggregates of the amyloid-β (Aβ) peptide cleaved from the amyloid precursor protein (APP) by β- and γ-secretases [7,8,9,10]. A massive loss of cholinergic neurons and decreased acetylcholine (ACh) levels are also hallmarks of the disease, resulting in attention deficits, memory impairment, and cognitive decline being major clinical manifestations of the disease [11,12].

Parkinson’s disease (PD) is characterized by degeneration and loss of dopaminergic neurons of substantia nigra pars compacta and the presence in neurons (of substantia nigra, cerebral cortex, locus coeruleus, dorsal nucleus of the vagus nerve, sympathetic ganglia, and the myenteric plexus of the intestine) of round, dense inclusion bodies, known as Lewy bodies, consisting mainly of misfolded α-synuclein, whose aggregation is linked to decreased neurotransmitter release and synaptic dysfunction [13,14]. The loss of dopaminergic neurons is connected to the major clinical symptoms of the disease such as tremors, rigidity, bradykinesia, and changed posture [15]. Non-motor clinical presentations of PD include various non-motor features such as autonomic dysfunction including gastrointestinal symptoms like constipation, cognitive and psychiatric changes, sensory symptoms, and sleep disturbances [13,15].

In addition to sharing oxidative stress, mitochondrial dysfunction, programmed cell death, and neuroinflammation as essential features of the two diseases [5,6,16,17], a common link between the gut microbiome and AD and PD, as well as between intestinal permeability and the two diseases, has also been established. A role of gut microbiota and compromised gut wall integrity in the pathogenesis and progression of the two diseases is increasingly acknowledged.

A well-known fact is that by synthesizing various neurotransmitters and neuromodulators, as well as by producing a wide variety of metabolites and using neural, immune, endocrine, and metabolic pathways, gut microbiota influence the brain and brain function [18]. This has been intensely researched and proven for food intake, satiety, obesity, and regulation of the gastrointestinal tract (e.g., [18,19,20]).

Recently, however, a growing body of evidence has been giving rise to the hypothesis that gut dysbiosis (leading to/accompanied by compromised gut wall integrity underlined by bacterial migration and inflammation) leads to activation of the peripheral immune response, altered production of neurotransmitters and bacterial metabolites, and a possible contribution to abnormal signaling through the vagus nerve (it is known that microbiota-related molecules can influence vagus nerve firing, and some can travel to the brain via the nerve [21]; also, neuropeptides, like cholecystokinin, for example, produced by endocrine cells in the gut can bind to specialized receptors on the vagus nerve [22]), leading to impaired blood–brain barrier function and an altered central immune response of microglia (but also of other brain cells), which promotes neuroinflammation and neuronal loss and contributes to the progression of AD or is implicated in its pathogenesis [23,24,25,26].

Only a few of the many supporting facts for the above hypothesis are as follows:

- Changes in the gut microbiome, correlating with the pathophysiology of AD, observed in both AD patients and mouse models of AD [24,25,27,28,29];
- Studies, using antibiotic treatment [30,31,32,33] or mice bred in germ-free conditions and devoid of gut microbiota [34,35,36], showing a strong reduction in Aβ pathology and in microglial activation in AD mice models;
- Evidence that gut dysbiosis may lead to raised levels of circulating gut microbiota metabolites, including β-N-methylamino-L-alanine and microbial amyloids, the former, a gut cyanobacteria-produced neurotoxin, shown to cause hippocampal cell death and learning and memory deficits [37,38,39];
- Studies on probiotic supplementation based on lactobacilli and bifidobacteria showing beneficial effects on cognitive function and other disease parameters in AD patients and animal models of AD and dementia [40,41,42,43].

Interestingly, Sun and colleagues show that AD could be initiated in the gut and only afterward spread to the brain. In their study, they administered Aβ1–42 oligomers into the serosa of the body of the stomach and the proximal colon of mice. The oligomers at 1 month were partly redistributed to the fundus and jejunum of the mice, and at 1 year, vagal and cerebral β-amyloidosis were observed; mice exhibited gastrointestinal dysfunction and cognitive deficits [44]. Thus, translocation of Aβ oligomers from the gut to the brain can significantly contribute to neuroinflammation and AD.

Regarding Parkinson’s disease, the hypothesis that the disease could start in the gut and spread to the brain [45] is even more beyond doubt. One of the most convincing pieces of evidence comes from the study of Holmqvist and colleagues [46] showing the propagation of α-synuclein retrogradely from the periphery to the brain. The authors injected the wall of the stomach and duodenum of rats with a substantia nigra lysate from a PD patient (confirmed to contain different α-synuclein forms) and demonstrated that α-synuclein from the injected lysate was taken up and transported via the vagal nerve to the brain. Even more, they confirmed their findings using labeled recombinant α-synuclein forms (monomers, oligomers, and fibrils), further strengthening the hypothesis that PD pathology may arise in the gut and gradually propagate to the brain, where it eventually could lead to PD. This is in line with Braak’s observations that Lewy pathology first manifests in enteric neurons of the gut long before it is present in dopaminergic neurons of the midbrain and PD symptoms are evident [47].

While the precise mechanisms by which the gastrointestinal system is involved in the pathogenesis (and possibly progression) of PD are yet to be fully elucidated, a role for gut dysbiosis and altered microenvironment of the gut, as well as for increased intestinal permeability in the pathogenesis of α-synucleinopathies and the pathophysiology of PD, seems more and more unquestionable. The topic is substantiated by a multitude of studies and covered in detail in a growing number of reviews. Changes in the gut microbiota and microbial metabolites initiate a pathological process in the gut, increasing the permeability of the blood–gut and blood–brain barriers, thereby providing a route of transmission between the contents of the gut and the brain, recognized as an accepted mechanism to drive neuroinflammation in the brain. Moreover, those changes induce the formation of α-synuclein pathogenic species, which propagate via the vagus nerve to the CNS where they induce other α-synucleins to undergo detrimental conformational changes in a prion-like manner and to form fibrils, which eventually accumulate in Lewy bodies and contribute to PD (for reviews, see, for example, [48,49,50,51]).

## 3. Zeolites

**Figure 1:**
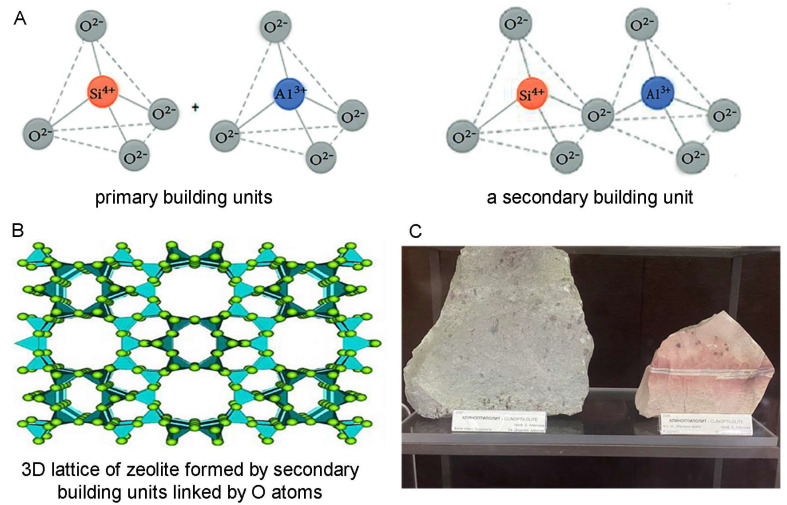
Structure of zeolites. (**A**) Primary and secondary building units of zeolite. The primary building units represent tetrahedral structures with mostly Si or Al in the center of the tetrahedron and O atoms at the corners (left), which are shown to be linking the tetrahedrons in the secondary units (right). (**B**) Secondary building units further linked by O atoms and forming a complex three-dimensional lattice underlying the microporous structure of zeolite holding cavities filled with water molecules and ions (by Derbe et al. [52]). (**C**) Clinoptilolites from the region of Kardzhali, Rhodope mountain, South Bulgaria (collection of the Museum “Earth and People”, Sofia, Bulgaria).

Zeolites are a large family of minerals built by [SiO_4_]^4−^ and [AlO_4_]^5−^ building blocks (tetrahedrons) joined by shared oxygen atoms and forming a three-dimensional microporous structure that encloses interconnected cavities holding water molecules and ions [52,53] (Figure 1A,B). In the zeolite framework, as Si is tetravalent and Al is trivalent, the SiO_4/2_ tetrahedrons are electroneutral, and the AlO_4/2_ tetrahedrons are negatively charged, the negative charge being compensated by mostly monovalent and divalent alkali and alkaline earth metal ions, such as sodium, potassium, and calcium residing in the zeolite cavities [54]. These cations can easily be exchanged with other cations from the surrounding environment, although zeolite interactions do not remain limited to cation exchange, as zeolites adsorb and interact with a range of different other ions and molecules as well (e.g., NH_4_^+^; e.g., [55]). Thus, zeolites are excellent ion-exchangers, adsorbents, and “molecular sieves” for sorting and separating various molecules, as well as catalysts, which, together with their thermal and chemical stability, including acid resistance, determines their numerous applications in industry for water, waste water, soil, and air purification; in oil refining; in the production of detergents; in chemistry, especially analytical chemistry; in agriculture; in livestock production; and many others [54,56,57,58,59].

The most widespread, studied, and applied zeolite is the naturally occurring zeolite clinoptilolite (Figure 1C). Additionally, mechanically transformed (modified) zeolite clinoptilolites such as micronized zeolite, tribomechanically activated zeolite (TMAZ), and double tribomechanically activated zeolite clinoptilolite (PMA zeolite), processed using a patented tribomechanical micronization technology (Panaceo Micro Activation), offer further improved beneficial effects of zeolite [60]. For their high capacity and efficiency as ion-exchangers and adsorbents and lack of detrimental physiological effects making them excellent detoxifying agents for animals and humans, clinoptilolite and, especially, its modified forms, micronized zeolite, TMAZ, and PMA zeolite, are of valuable medicinal use as well. Furthermore, clinoptilolite and its forms possess antioxidant, anti-inflammatory, and immunomodulatory properties and exert positive effects on gut barrier function, gut microbiota, and intestinal health, all of which define their place in a multitargeted treatment of neurodegenerative diseases and especially AD and PD.

## 4. Zeolite Effects on Gut Microbiota and Function

Zeolite has been shown to influence intestinal health in a number of ways, most of them probably interconnected. It has a direct beneficial effect on the microbial population of the intestine. Wu et al. demonstrated that supplementation of the diet of chickens with natural and modified clinoptilolite significantly decreased the total counts of viable *E. coli* and significantly increased those of Lactobacillus acidophilus [58]. Furthermore, without reducing the microbial richness and diversity of the gut, in laying hens, natural zeolite has been shown to decrease the load of bacteria within the phylum Proteobacteria, especially the family Enterobacteriaceae, known to be associated with chronic dysbiosis, obesity, inflammatory bowel disease, ulcerative colitis, Crohn’s disease, and other health conditions [61,62]. In turn, Lactobacillus and Bifidobacterium are increased with a simultaneous decrease in the number of Klebsiella and Enterobacter in dogs fed on chabazitic zeolite [63]. In humans, although not statistically significant, an increase in Bifidobacteria and Lactobacillus species and a reduction in Firmicutes species were observed under treatment with PMA zeolite as part of a therapy for irritable bowel syndrome [64].

Numerous studies in poultry, pigs, and cattle have shown that when used as a dietary supplement, zeolite exerts beneficial effects on gut development [57], gut morphology (by influencing intestinal parameters such as villus height, villus perimeter, villus section area, crypt depth, and villus height to crypt depth ratio and thus having a positive effect on the nutrient absorption and digestive capacity of the small intestine) [58,65,66], and gut digestive enzyme activity [58,65]. Furthermore, zeolite has been shown to lower small intestine and cecal pH values [58], and weakly acidic pH conditions in the intestinal tract have been shown to contribute significantly to the absorption of vitamins, electrolytes, and iron; to the activation of digestive enzymes; and to the inhibition of the growth of pathogenic bacteria, as well as to many other beneficial effects (for a review, see [67]). Reaching even further, as zeolite supplementation exerts similar or better effects than antibiotic dietary inclusion, clinoptilolite has been suggested as antibiotic substitute therapy in livestock production and for the treatment of gastrointestinal problems [66,68]. Indeed, zeolite efficiently works against Gram-negative bacteria [58,63,69,70], including *E. coli*, which carries certain antimicrobials resistance genes [70], and reduces the abundancies of antibiotic resistance genes (ARGs) in vitro and in vivo [66,71].

Particularly important, however, are the effects of zeolite on gut wall integrity and gut barrier function, especially since increased intestinal permeability has been observed in PD and AD patients. Indeed, not only has increased intestinal permeability been observed in PD patients, but it has been strongly correlated with excessive endotoxin exposure (as judged by increased intensity of staining for *E. coli* in both epithelial and lamina propria zones of sigmoid mucosa samples and lower levels of lipopolysaccharides binding protein (LBP), associated with increased exposure to Gram-negative bacteria), increased oxidative stress (increased intestinal staining for nitrotyrosine), and abnormal accumulation of α-synuclein in enteric neurons (α-synuclein staining in the intestinal mucosa) [72]. Immunohistochemical staining of postmortem samples of intestines of PD patients also exhibits an association between impaired intestinal barrier integrity, an increase in the intestinal bacterial flora, high levels of expression of inflammatory genes, and abnormal accumulation of α-synuclein in the enteric nervous system [73]. A recent study shows that AD model mice exhibit excessive Aβ deposition in the intestinal epithelium, accompanied by decreased intestinal concentrations of tight junction proteins such as occludin, claudin-1, and Zonula occludens-1; disruption of the gut barrier integrity; and greater intestinal permeability, as well as gut inflammatory changes [74]. Disturbed intestinal barrier function has been observed in other AD mouse models, as well as in AD patients [75,76,77].

Selective permeability through the intestinal epithelium is transcellular (across epithelial cells) and paracellular (through the spaces between epithelial cells) [78]. Tight junctions, multiprotein complexes (comprising at least 40 different proteins, among which are transmembrane proteins like claudins, occludin, and junctional adhesion molecules (JAMs) but also intracellular peripheral membrane proteins that form a scaffold between the transmembrane proteins and the actin cytoskeleton such as Zonula occludens-1 (ZO-1), ZO-2, and ZO-3) that “seal” adjacent epithelial cells, are a major determinant of paracellular permeability and a key factor in the “leaky gut” condition as increased permeability of gut mucosa is often referred to [79,80,81,82,83]. Zonulin is acknowledged as the main physiological modulator of intercellular tight junctions, changing the tight junction competency, driving “opening”of the paracellular transport pathway, and increasing both intestinal and gastroduodenal permeability [84]. Alpha1-antitrypsin is considered another marker of intestinal barrier integrity, and its concentration in the feces is widely used to estimate protein leakage into the intestinal tract, as well as intestinal inflammation [85,86,87]. Furthermore, plasma levels of D (-) lactate, a product of bacterial fermentation of carbohydrates, and bacterial lipopolysaccharides (LPSs), the major constituent of the outer membranes of Gram-negative bacteria (e.g., *E. coli*) and a potent bacterial endotoxin, together with diamine oxidase (DAO) activity, reflect intestinal barrier function and are used for monitoring intestinal permeability. The reduction in DAO activity and blood levels of D-lactic acid and LPSs generally correlate with improved intestinal barrier function [88,89]. In chickens, dietary zeolite supplementation significantly decreased LPS concentrations, D-lactic acid levels, and DAO activity and upregulated ZO-1, occludin, and claudin mRNA abundance in duodenal mucosa [66,90], as well as claudin in cecal mucosa.

In humans, two studies show favorable effects of zeolite on intestinal wall integrity and function [64,91]. Based on data showing that in performance sports, there is a high prevalence of gastrointestinal complaints accompanied by increased intestinal wall permeability, Lamprecht and colleagues demonstrated that in endurance-trained men and women, 12 weeks of zeolite supplementation (PMA zeolite (PANACEO SPORT^®^,Panaceo International Active Mineral Production GmbH, Villach, Austria)) decreased zonulin accompanied by mild anti-inflammatory effects [91]. Petkov and colleagues showed that in patients with irritable bowel syndrome, PMA zeolite significantly decreased alpha1-antitrypsin, paralleled by lowered levels of the inflammation marker hsCRP (high sensitive C-Reactive Protein), increases in Bifidobacteria and Lactobacillus species, and reductions in Firmicutes species [64].

Although the detailed mechanisms by which zeolite strenghtens the intestinal barrier and improves gut health are unclear, they are, uncontroversially, related to its favorable modulation of the gut microbiota, as well as to its powerful detoxifying, antioxidative, anti-inflammatory, and immunostimulatory effects.

## 5. Detoxifying Effects of Zeolite

Due to their unique microporous structure and physical and chemical properties, defining their exceptional ion-exchange and adsorption capacity, zeolite and its modifications serve as excellent detoxifying agents. The detoxifying effects of zeolite, micronized zeolite, TMAZ, and PMA zeolite cover a wide range of harmful and toxic substances of inorganic and organic origins, as well as heavy metals.

### 5.1. Detoxifying Effects of Zeolite-Ammonium, Nitrates, Nitrites, Mycotoxins, Pesticides, Synthetic Neurotoxins, and Radionuclides

Diets rich in proteins and with reduced carbohydrate content modify the colonic microbiome, favoring a potentially pathogenic and pro-inflammatory microbiota profile, decreasing short-chain fatty acid production, and increasing ammonia and hydrogen sulphide concentrations, the latter, reflecting excessive protein fermentation, being implicated in the pathogenesis of irritable bowel syndrome, ulcerative colitis, and colorectal carcinogenesis as well [92]. Zeolite has a proven very high affinity toward ammonium (the selectivity of ion exchange on natural clinoptilolite has been reported in an order of K^+^ > NH_4_ ^+^ > Na^+^ > Ca^2+^ > Mg^2+^ (e.g., [93,94])) and has long been used to efficiently remove ammonium from fresh and waste water [55,95,96,97,98] and also shows favorable effects in humans when used as a supplement in the protein-rich diet of endurance-trained individuals [91]. Except for ammonium (NH_4_^+^), zeolite has shown high removal efficiency for nitrates (NO_3_^−^) and nitrites (NO_2_^−^) as well [55,99]. A study performed by Katsoulis and colleagues on dairy cattle showed that the prolonged consumption of water with increased nitrate levels impaired protein metabolism and glucose utilization, while the dietary administration of clinoptilolite relieved the nitrates’ effects [100].

Furthermore, zeolite, synthetic zeolite, and nanozeolite are effective in retaining and/or fighting the effects of a wide range of mycotoxins like zearalenone, ochratoxin A, deoxynivalenol, and especially the widespread aflatoxins [101,102,103,104,105,106,107]. Aflatoxins are toxic metabolites produced by the Aspergillus species, and their principal biological effects are carcinogenicity, immunosuppression, mutagenicity, and teratogenicity [108]. Animal feeds are highly susceptible to mycotoxins’ (including aflatoxins’) contamination during harvest, storage, and processing, and mycotoxicoses, the result of consuming feeds contaminated with mycotoxins, have highly detrimental effects on animal health and production [104]. Aflatoxin residues might occur in milk and other animal products and be carried over in products used for human consumption [105].

Zeolite also absorbs and/or alleviates the consequences of other highly toxic and harmful substances such as pesticides like dichlorvos [109], synthetic neurotoxins like VX belonging to the organophosphorus class [110,111], and radionuclides [112,113].

### 5.2. Detoxifying Effects of Zeolite-Heavy Metals

Of special importance is the potential of zeolites for removal of heavy metals that present a serious risk to human health as they do not disintegrate and cannot be eliminated from the human body by metabolic processes [114]. Heavy metals are generally defined as the metals with relatively high atomic weights (63.5–200.6 g/mol) and of densities above 5 g/cm^3^ [115]. Although the body is in demand of low amounts of some heavy metal ions such as Zn^2+^, Mn^2+^, Fe^2+^, and Cu^2+^ for growth and body health, high levels of those are toxic [116]. Other heavy metals including Pb, Cd, Hg, As, and Cr, even at low doses, are considered extremely harmful, although their levels in the environment resulting from heavy industry, transport, mining, and agriculture are continuously rising [117]. Studies show that even low-level exposure, but simultaneously, to Pb, Cd, and Hg causes damage to multiple organs in rats, as well as impairments in neurobehavioral functions such as impairments in learning and memory, as well as sensory perception and histopathological changes in the brain, liver, kidney, and testicle [118].

Zeolites, being highly efficient “molecular sieves”, show enormous capacity to eliminate harmful metals, and this property has been utilized in both animals and humans, especially since it is manifested not at the expense of removing physiologically relevant elements, i.e., micronutrients and trace elements [119]. Selectivity for clinoptilolite cation exchange in the order Pb^2+^ > Cd^2+^ > Cs^+^ > Cu^2+^ > Co^2+^ > Cr^3+^ > Zn^2+^ > Ni^2+^ > Hg^2+^ [120] makes it highly useful in lead poisoning. Indeed, in lead poisoned mice, the clinoptilolite sorbent KLS-10-MA, a modified natural Bulgarian zeolite, reduced the accumulation of Pb by 84%, 89%, 91%, 77%, and 88% in carcass, liver, kidneys, bones, and feces of mice, respectively [121], and plentiful studies demonstrate that the Pb intoxication burden in animals can be alleviated by food clinoptilolite supplementation [122,123,124]. In rats, Nikpey and colleagues have demonstrated the protective effect of microporous natural clinoptilolite on lead-induced learning and memory impairments [125]. Zeolites are equally efficient against Cd toxicity. For example, it has been shown that clinoptilolite supplementation prevented Cd-induced Fe-deficient anemia [126]. Although zeolites are especially effective in removing Pb and Cd, they show high potential, proven in the literature, also for chromium (Cr), arsenic (As), manganese (Mn), zinc (Zn), nickel (Ni), and mercury (Hg) [114,127]. Zeolite is an excellent sorbent also for cobalt (Co), iron (Fe), and especially copper (Cu) [128,129,130,131]. Chronic excessive exposure to aluminium (Al), although the element is not classified as a heavy metal, may result in oxidative stress, immunological alterations, proinflammatory effects, peptide denaturation or transformation, enzymatic dysfunction, metabolic derangement, amyloidogenesis, membrane perturbations, iron dyshomeostasis, apoptosis and dysplasia, and a multitude of pathological conditions, some of which include inflammatory bowel disease, Crohn’s disease, anemia, pancreatitis, diabetes mellitus, toxic myocarditis, thrombosis and ischemic stroke, and autism, as well as dementia and Alzheimer’s disease [132]. Data in the literature show the excellent properties of PMA zeolite with increased active surface and activity compared with natural clinoptilolite in Al elimination from the body [114,133].

### 5.3. Heavy Metals and Neurodegenerative Diseases

Heavy metals are involved in various pathophysiological mechanisms associated with neurodegenerative diseases including Alzheimer’s disease, Parkinson’s disease, Huntington’s disease, multiple sclerosis, and amyotrophic lateral sclerosis (ALS) [134,135,136,137].

**Lead (Pb) and cadmium (Cd)** are overt neurotoxins, and effects through oxidative stress, neuroinflammation, mitochondrial dysfunction, and ultimately neuronal apoptosis are well defined. Cadmium may also induce neurotoxicity by changing the permeability of the blood–brain barrier and interacting with other neurotoxins, leading to Aβ aggregation and NFT production [138].

In animal models, treatment with Pb, especially at an early age, results in elevated brain levels of APP, Aβ, and tau, as well as impairments in learning and memory [138,139]. A most impressive example is the study on primates exposed to Pb as infants and the manifested increases in the expression of APP, β-secretase, and Aβ, as well as higher levels of oxidative damage to DNA and a decrease in DNA methyltransferase activity accompanied by enhanced AD pathology, in the aged animals [140]. In humans, consistent long-term studies or observations on the effect of early life Pb exposure in relation to AD are generally not sufficient [138,141], but in older adults, it has been demonstrated that lead exposure is associated with lower cognitive status and longitudinal declines in cognition [138]. Furthermore, in workers with occupational exposure to Pb, impairments in verbal and visual memory performances have been demonstrated [134].

Cd is increasingly recognized as an environmental factor for both AD and PD [142,143]. Animal studies support a link between cadmium and Aβ aggregation and NFT accumulation and aggravation of AD pathology at Cd exposure [138,144,145]. In humans, in a meta-analysis and systematic review, Xu and colleagues show significantly higher circulatory levels of Cd in AD patients compared with controls, whereas in another review, Bakulski and colleagues reveal that cadmium may be associated with decreased cognitive function and clinical AD specifically [138,146]. In a case study it has been reported that a 64-year-old man who suffered from acute exposure to cadmium developed parkinsonian features [143].

In a healthy brain, **Fe, Cu, Mn, and Zn** play fundamental roles as enzyme cofactors, in support of neurotransmission, cellular metabolism, and the antioxidant defense. Imbalances in these heavy metals and especially increases in their levels can lead to oxidative stress and reactive oxygen species generation, protein aggregation, neuroinflammation, and mitochondrial dysfunction (thereby contributing to neurodegeneration) or directly exacerbate the AD and PD pathology [134,135,136,137].

**Iron (Fe)** is essential for the proper physiological functions of living organisms, being an indispensable part of hemoglobin and a number of important proteins and enzymes such as cytochromes and catalases, necessary for oxygen and energy metabolism and the antioxidant defense, as well as other vital processes [147]. Non-physiological increases in Fe in brain regions of AD patients (including the hippocampus, substantia nigra, globus pallidum, putamen, and caudate nucleus) [148] along with robust evidence for a role of Fe in Aβ aggregation and toxicity, for its enrichment in senile plaques and NFTs and for furthering AD pathology (including accelerating the aggregation of tau proteins), have been reported [149,150,151,152]. For example, it has been shown that Fe promotes the toxicity of the amyloid beta peptide by impeding its ordered aggregation [149]. Increased Fe accumulation has been observed in the brain of PD patients as well, especially in substantia nigra, defined in vivo and in postmortem studies [135,153,154,155,156,157], and Fe has been shown to increase the aggregation of α-synuclein [158,159,160,161]. Increases in Fe in the brain have been found in multiple sclerosis and Huntington’s disease patients as well [162,163].

**Manganese (Mn)** in low concentrations is important as it serves as a cofactor for a variety of enzymes such as Mn superoxide dismutase, arginase, glutamine synthetase, various hydrolases, like agmatinase for example, and lyases [164,165]. Although it has been demonstrated in animal models that excessive manganese causes AD pathology including Aβ accumulation and tau phosphorylation and in human epidemiologic studies that it binds Aβ and is related to cognitive decline [138], Mn overexposure has been mostly recognized for a type of parkinsonism it causes known as manganism. Some evidence suggests that Mn could have some importance in the early stages of Huntington’s disease, but it remains unclear whether it plays a role in the actual onset of the disease. Regarding ALS, magnetic resonance imaging studies of a number of ALS patients show neurological changes characteristic for manganese overload, although precise quantification of Mn levels in ALS patients is not available [137]. 

In both AD and PD, disorders in Cu^2+^ and Zn^2+^ homeostasis play a pivotal role in the mechanisms of their pathogenesis [135].

**Copper (Cu)** is required for important cellular processes such as respiration, defense against oxidative stress, neural transmission, iron metabolism, and protein modification, acting as a cofactor to enzymes like cytochrome c oxidase, Cu/Zn-superoxide dismutase, dopamine β hydroxylase, hephaestin, ceruloplasmin, lysyl oxidase, and amine oxidases [166]. However, excessive Cu amounts in the organism are toxic and are associated with AD, PD, ALS, and Huntington’s disease [134,163] Of all the physiologically relevant metals, Fe, Cu, Mn, and Zn, copper excess has been shown to be most consistently related to AD. Thus, a recent review by Babić Leko and colleagues [167] examined the literature for metals like Al, As, Ba, Co, Cu, Cd, Ca, Fe, Li, Pb, Hg, Mg, Mo, Mn, Ni, K, Se, Na, Sr, Tl, and Zn and (1) compared their concentrations between AD patients and healthy controls, (2) correlated concentrations of AD cerebrospinal fluid (CSF) biomarkers with metal concentrations, and (3) used Mendelian randomization to assess the potential metal contributions to AD risk. Babić Leko and colleagues concluded that the most consistent findings were for Zn and Cu, with most studies observing a decrease in Zn levels and an increase in Cu levels in AD patients. In addition, there is a study correlating differences in the Cu levels in the blood, CSF, and brain of AD patients with the development of cognitive impairments and the transition between phases of the AD-related spectrum [168]. Indeed, it has been shown that Cu is elevated in senile plaques and in NFTs and appears to promote both the progression of the Aβ cascade and the formation of the neurofibrillary tangles (being reported to bind to the hyperphosphorylated tau protein before the formation of intracellular tangles) [169,170,171]. In line, it has been shown that copper binds the amyloid precursor protein in its extracellular domain [172], and a high-affinity interaction is carried on also with the Aβ peptide through a histidine-binding motif [173]. Furthermore, in an AD mouse model, exposure to high levels of Cu in the drinking water increased Aβ and tau buildup by upregulating the activitity of β-secretase and by activating cdk5/p25 acompanied by accelerated cognitive impairment [174,175]. Interestingly, it has also been demonstrated that Aβ [176] and APP [172] could reduce Cu^2+^ to Cu^+^, which in turn can catalyze the decomposition of hydrogen peroxide via the Fenton reaction,

Cu^+^ (Fe^2+^) + H_2_O_2_ → Cu^2+^ (Fe^3+^) + •OH + OH^−^,

generating reactive hydroxyl radicals (•OH), capable of damaging DNA, lipids, and proteins and further contributing to the AD pathology [137]. Regarding Parkinson’s disease, it has been demonstrated that excess Cu leads to neuronal cell death and promotes α-synuclein aggregation [177,178,179].

**Aluminium (Al)** is the third most abundant element and widely in use in everyday life. Environmental exposure, food, cosmetics, and medicinal products like, for example, vaccines (where Al salts are used as adjuvants) are sources of Al intake in the body including brain absorption as Al crosses the blood–brain barrier [134]. Al has no physiological function whatsoever and is an accepted neurotoxin. Al neurotoxicity is especially affecting memory and cognitive functions, and a link between Al exposure and AD has been postulated since 1965 [180], widely discussed and supported in numerous research studies and reviews since (e.g., [132,181,182]). For example, Al influences acetyl-CoA and inhibits acetylcholine release [183,184], interferes with hippocampal calcium signaling important for synaptic plasticity [185], impairs hippocampal long-term potentiation in rats in vitro and in vivo [186], and causes learning disorders and memory deficits in experimental animals (e.g., [187]). In direct relation to AD, results from a meta-analysis study show that individuals chronically exposed to Al are 71% more likely to develop AD [188]. Indeed, highly sensitive techniques like laser microprobe mass analysis (LAMMA) and energy-dispersive X-ray spectroscopy combined with transmission electron microscopy (TEMEDX) have demonstrated an accumulation of Al in both senile plaques and NFTs of AD patients [152,189]. In addition, Al inhibits proteolytic degradation of Aβ and enhances its polymerization and the formation of stable Aβ oligomers [150,190,191]. The Al-induced oligomerization is more marked than that induced by other metals like Zn^2+^, Fe^3+^, Cu^2+^, and Cd^2+^. Al-aggregated Aβs have a stronger affinity for membrane surfaces as a result of minimal degradation by proteases, and Al–Aβ complexes are more toxic than Aβs alone causing membrane disruption or disturbances in neural Ca^2+^ homeostasis and mitochondrial respiration [181]. Al accelerates also the phosphorylation of tau and its accumulation, as well as inhibits its dephosphorylation [181]. It induces an accumulation of hyperphosphorylated tau, increases Aβ levels, and accelerates Aβ plaque deposition in experimental animals [192,193]. Al has been associated also with ALS and multiple sclerosis [194,195].

## 6. Anti-Inflammatory, Antiapoptotic, and Immunomodulatory Effects of Zeolite

Zeolite exerts convincing anti-inflammatory and positive immunomodulatory effects.

### 6.1. Anti-Inflammatory and Antiapoptotic Effects of Zeolite

LPSs, produced by Gram-negative bacteria and being powerful endotoxins, elicit strong gut inflammatory responses and induce a release in the host of an array of proinflammatory cytokines, among which tumor necrosis factor alpha (TNF-α), IL-1β, IL-6, IL-8, and IL-12 are the major mediators [196,197]. In LPS-challenged chickens that were pretreated with natural or modified clinoptilolite, the LPS-induced increase in the plasma and intestinal mucosa concentrations of TNF-α, IL-1β, IL-2, IL-6, and IL-4 was dramatically attenuated in the clinoptilolite-pretreated groups [90]. Nuclear factor-kappa B (NF-κB) is a ubiquitous transcription factor regulating the expression of numerous genes, most of which are involved in inflammatory and immune responses but are also pertinent to cell survival, proliferation, and differentiation. Two signaling pathways are involved in NF-κB activation, and the classical, more common, pathway is triggered by inflammatory cytokines like TNF-α, IL-1, IL-2, IL-17, interferones, toll-like receptors, T- and B-cell receptor signals, and other stimuli. A large number of studies implicate NF-κB in the pathogenesis of tumors, as well also for its role in promoting the production of chemokines, growth factors, and cytokines and other molecules that constitute the tumor microenvironment [198]. Clinoptilolite has been demonstrated to counteract the inflammatory and apoptotic effects of adriamycin, an anthracycline drug used as a chemotherapy medication, by decreasing TNF-α, IL-1β, and NF-κB levels in liver cell cultures. Furthermore, using artificial neural network models, Krajisnik and colleagues show that the anti-inflammatory effects induced by diclofenac sodium are intensified and prolonged in the presence of zeolite [199].

In humans, although not reaching statistical significance, PMA zeolite supplementation (PANACEO SPORT^®^) has been shown to increase the level of the anti-inflammatory cytokine IL-10. As stated by the authors, such a tendential increase was possibly reflecting anti-inflammatory modulation, which could be linked to the reduced intestinal wall permeability in the zeolite group as indicated by significant zonulin decrease [91]. Interestingly, the protective capability of probiotics, like, for example, L. salivarius Ls33, correlates with local IL-10 production [200]. Petkov and colleagues, in the patients with irritable bowel syndrome, also showed anti-inflammatory effects of PMA zeolite through lowered levels of the highly sensitive C-Reactive Protein [64].

### 6.2. Immunomodulatory Effects of Zeolite

Evidence is accumulating that zeolites may significantly affect the regulation of the immune system and act as immunostimulators.

The gut immune response is carried out by the gut-associated lymphoid tissue (GALT), consisting of major structures such as the Peyer’s patches and the mesenteric lymph nodes, as well as isolated lymphoid follicles [201]. Peyer’s patches are initiation, relay, and effector centers sensing and trapping antigens from gut microbiota and foreign particles in the intestinal lumen and organizing and managing their destruction in a process of immune surveillance and the gut mucosal immune response. The epithelium of the Peyer’s patches is devoid of Goblet cells and thus not covered by mucus and is characterized by the presence of microfold (M) cells, which sample and ingest antigens and deliver them to antigen-presenting cells such as dendritic cells and macrophages, which further triggers specific T- and B-cell responses [201]. GALT hosts a large number of immune cells such as dendritic cells, macrophages, different T lymphocytes (including Treg or helper cells Th1, Th2, and Th17 and follicular T helper cells (Tfh)), and B cells such as plasma cells, the latter producing high levels of specific secretory immunoglobulin A (sIgA). sIgAs are major players in shaping the intestinal immune response as they bind both to symbiotic bacteria controlling their colonization of the gut and to pathogenic bacteria helping the development of immune tolerance and at the same time defending against foreign invaders [202,203]. A recent meta-analysis study assessing the effects of clay mineral supplementation on alkaline phosphatase and broiler health and performance, based on 369 research items from 86 studies, showed that zeolite increased significantly the body weight, average daily gain, and performance efficiency index of chickens, when in their starter phase, along with significantly increased concentrations of IgA and IgM in the jejunum and ileum of the chickens [204]. Additionally, when supplemented in the food of chickens, Zn-bearing clinoptilolite [205] and a mixture of zeolite and attapulgite [206] also increased IgA in the jejunum of chickens. In line, Jarosz and colleagues report an increased percentage of B lymphocytes in chickens fed on 2% Zakarpacki zeolite [207]. Positive immunomodulatory effects have been reported in other studies as well, with clinoptilolite and ZnO supported on zeolite in pigs [208,209] and in humans [210]. In patients with immunodeficiency disorder, Ivcovic and colleagues reported that TMAZ supplementation produced significant and relevant increases in B lymphocytes (CD19+), Th cells (CD4+), and activated T lymphocytes (HLA-DR+) accompanied by reported improved well-being of the patients [210].

Pavelic and colleagues propose that the immunostimulatory effects of zeolite could be partly due to its interaction with the M cells of the intestinal epithilium of Peyer’s patches. They hypothesize that in a difference from other cells of the intestine with a rich negatively charged glycocalix, M cells, which lack such a glycocalix, could absorb the negatively charged clinoptilolite paricles, transport them across the intestinal barrier, and present them to Ag-presenting cells in the Peyer’s patches modulating the immune response without entering the circulation [119]. In support, bulk SiO_2_ was found to be transported by M cells [211]. Zeolite has also been suggested to act as a non-specific immunostimulator in a manner similar to that of the superantigens (SAgs), which, unlike conventional antigenic peptides that activate 0.0001–0.001% of the organism T cells, recruit up to 10–30% of the T-cell repertoire [119,210].

## 7. Antioxidative Properties of Zeolite

Oxidative stress, defined as the imbalance between oxidant molecules and the organism’s antioxidant defence system and reflected in increased lipid peroxidation, generation of excessive free radicals, and altered antioxidant enzyme activities, has been shown to be related to impairments in learning and memory and is considered part of the pathophysiological mechanism involved in the progression of cognitive dysfunction [212,213,214]. Oxidative damage along with accompanying enhanced lipid peroxidation and decreased activity of the major antioxidant enzymes such as superoxide dismutase (SOD), catalase (CAT), and glutathione peroxidase (GPx), as well as decreased levels of glutathione (GSH), have been shown to be characteristic features of AD and PD as well [215,216,217].

Zeolites exhibit strong antioxidant activity that has been shown in vitro, in animal studies, as well as in human trials.

Zeolites have been shown as effective ROS scavangers in vitro [218,219,220]. Thus, in an in vitro experimental model of mitochondrial ROS production (human neuroblastoma SH-SY5Y cells exposed to progressively increasing laser intensity), PMA zeolite pretreatment induced a significant reduction in mitochondrial ROS and cell death [219]. When zeolites were used to eliminate the effects of ROS (namely, •OH) on human albumin under in vitro conditions, no saturation of the respective zeolite active sites was observed (possibly because of active conversion of the hydroxyl radical to water molecules within the zeolite void system) [220].

In chickens, clinoptilolite and modified clinoptilolite were shown to increase the activities of liver CAT, GPx, total SOD, and the total antioxidant capacity, as well as to decrease malondialdehyde (MDA) levels as a measure of lipid peroxidation and the activity of total nitric oxide synthase [221]. Similar results were also shown for Zn-bearing clinoptilolite (ZnCP) in Salmonella pullorum-challenged chickens. ZnCP reduced the MDA content of jejunal and ileal mucosa and improved the SOD activity of ileal mucosa in the Salmonella pullorum-infected group of chickens [222]. The antioxidative effects of zeolite and its modified forms are also very convincing in mouse and rat models of different conditions accompanied by significant oxidative stress. In a dedicated study in Pb-exposed developing mice, Basha and colleagues aimed to elucidate the ameliorating effects of clinoptilolite especially on oxidative stress parameters [123]. As stated by the authors, Pb-intoxicated mice showed severe oxidative stress reflected in increased lipid peroxidation and decreased CAT, SOD, and GPx activities and GSH levels in a number of brain regions such as the cortex, cerebellum, brainstem, and hippocampus. Clinoptilolite lowered the lipid peroxidation levels and augmented CAT, SOD, and GPx activities in different brain regions indicating significant recovery from Pb-induced oxidative stress. Likewise, in hepatectomized rats, in which due to the trauma, oxidative stress markers were induced, plasma and liver tissue MDA levels were significantly lower in the micronized clinoptilolite-treated group compared with the control group and liver tissue Cu-Zn SOD activity, and GSH levels were significantly higher in the micronized clinoptilolite-treated group compared with the control group [223]. Last but not least, an increase in the SOD activity in the hippocampus of an AD model mouse when treated with PMA zeolite was also observed [219].

Clinical studies in humans also demonstrate that orally administered zeolites are potent antioxidants. Thus, in healthy volunteers, in both a smoker and a non-smoker group, Dogliotti and colleagues demonstrated that natural zeolites, such as chabazite, phillipsite, and analcime, administered perorally for 4 weeks, increased the blood levels of gluthatione peroxidase, superoxide dismutase, and gluthatione reductase and reduced lipid peroxidation [224]. Next, in a randomized, double-blind clinical trial on smokers, it was further shown that micronized and activated natural zeolite increased catalase activity and decreased H_2_O_2_ levels accompanied by a reduction in lipid peroxidation [225].

The mechanism by which zeolites exert their antioxidant effect is as of yet unclear, but one explanation, additional to directly trapping ROS, could possibly be assigned to the ability of zeolites to avail metal ions present within their structure as cofactors for the activation of the organism antioxidant enzymes [60].

## 8. Direct Effects of Zeolite on AD Pathology

While through its positive effects on intestinal health through its detoxifying, anti-inflammatory, and antioxidative properties, zeolite is strongly implied and indicated for the treatment of neurodegenerative diseases, especially AD and PD, some direct evidence for the effect of zeolite on AD pathology are also available.

APPswePS1dE9 transgenic mice develop Aβ deposits in the brain by 6 to 7 months of age [226] and are useful models for studying aging, amyloid plaque formation, and Alzheimer’s disease. Using APPswePS1dE9 transgenic mice and after a 5-month period of double tribomechanically activated zeolite supplementation in the drinking water of the animals, Montinaro and colleagues reported a significant 54% reduction in Aβ42 total levels in the hippocampus of the treated animals and about a 60% and 56% reduction in both the plaque number and amyloid load, respectively, in the mouse brains [219]. As the changes in Aβ42 levels and plaque load were paralleled by significant increases in SOD activity and by post-translational oxidative protein nitration in the hippocampus of zeolite-treated animals, and by reduced mitochondrial ROS production and accumulation in vitro, the authors attributed the beneficial AD effects in the mice to the zeolite antioxidant effects.

Very interesting is a recent study by Omran and colleagues performed with an AlCl_3_-induced rat AD model [227]. The authors showed that rats treated for 5 weeks with AlCl_3_ exhibited increased acetylcholinesterase (AChE) activity and Aβ and TNF-α levels, as well as lipid peroxidation (increased MDA levels), accompanied by learning and memory deficits as demonstrated by changes in behavioral experiments like the Morris water maze (MWM) and the passive avoidance test.

They showed AlCl_3_-induced changes in other parameters related to AD as well, like decreases in the brain-derived neurotrophic factor (BDNF) and the phosphorylated glycogen synthase kinase-3β (P-GSK-3β) content and increases in the NF-κB content [227].

Even though, as described in a previous section of this Discussion, better known for its role in inflammation, NF-κB has been implicated in synaptic plasticity, learning, and memory as well, and its expression and/or activation have mostly been shown to be increased in AD patients [228]. Regarding GSK-3β, attenuation of the phosphatidylinositol 3-kinases/Akt (PI3K/Akt) pathway, as reported in AD, causes GSK-3β activity increase leading to Tau hyperphosphorylation and progression of AD [229]. Under non-pathological conditions, an active Akt phosphorylates GSK-3β at Ser 9 and renders it inactive, making high levels of phosphorylated GSK-3β (P-GSK-3β) indicative for a beneficial effect on AD. Low BDNF mRNA and protein levels in postmortem AD brain samples have been shown, as well as a protective effect of BDNF against Aβ-induced neurotoxicity in vitro and in vivo in rats [230,231].

In their study, Omran and colleagues showed that zeolite significantly decreased the AlCl_3_-induced increases in AChE activity and Aβ, TNF-α, and NF-κB levels, as well as lipid peroxidation. Aditionally, it increased the AlCl_3_-decreased BDNF and P-GSK-3β levels, accompanied by counteraction of the neuronal damage in the hippocampus and striatum of the AlCl_3_-treated mice and by improvement in the MWM and passive avoidance tests parameters.

The positive effects of zeolite on AD pathology have been further confirmed in vitro as well. Lucas and Keitz studied the influence of zeolites on Aβ aggregation by exposing Aβ1-40 to zeolite Y containing different metal cations, including Na^+^, Mg^2+^, Fe^3+^, Zn^2+^, and Cu^2+^. Intriguingly, the Aβ1-40 aggregation turned very dependent on the metal cation. While NaY, MgY, and FeY accelerated the kinetics of fibrilization, CuY and ZnY, which showed higher association constants than the other zeolites, inhibited fibrilization. Further on, Western blots confirmed that Aβ1-40 was arrested at the oligomeric stage in the presence of ZnY and CuY, while continuing to the fibrillary state in the presence of the other zeolites [232].

In another study it was shown that zeolite nanoparticles inhibited the interaction between Aβ and fibrinogen, which leads to the formation of structurally abnormal clots, resistant to lysis, in the cerebral vessels of patients with AD. Such an inhibitory effect of the zeolite nanoparticles, with no significant effect on the regular clot formation and lysis hemostasis, is of further beneficial value as, in addition to the abnormal clots, the Aβ−fibrinogen interaction increases vascular Aβ deposition, affecting the blood−brain barrier function and advancing the cognitive impairments characteristic of AD [233].

**Figure 2:**
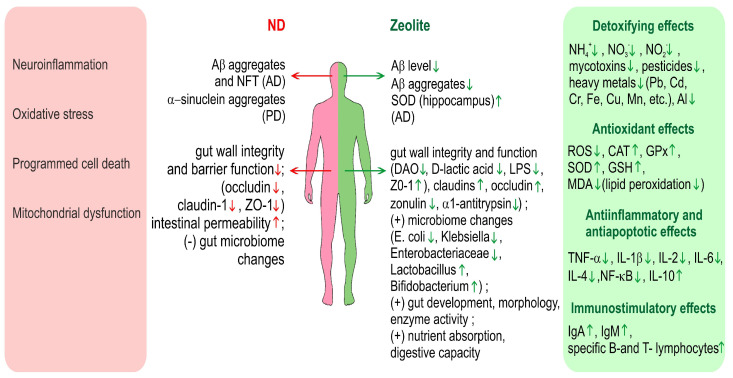
Overview on the various effects of zeolite in relation to neurodegenerative diseases and especially AD and PD. Green arrows denote positive effects and red arrows-negative. ND—neurodegenerative diseases, DAO—diamine oxidase, ZO-1—Zonula occludens-1.

Figure 2 gives an overview on the various effects of zeolite in relation to neurodegenerative diseases and especially AD and PD.

## 9. Zeolites as Biosensors and Drug Delivery Systems for PD

Zeolite and zeolite-based materials are relevant not only for the treatment of neurodegenerative diseases such as AD but also for various other related applications such as biosensors and drug delivery systems. Due to its molecular sieve selectivity, its cation-exchange capacity, and inherent catalytic properties, zeolite is finding numerous applications in electroanalysis and especially in “chemically modified electrodes” (CMEs) [234,235]. Voltammetric biosensors based on zeolite-modified electrodes have been developed for the determination of phenols, herbicides, and glucose, as well as L-DOPA and dopamine [59,236,237]. In view of the importance of dopamine not only for PD but also for attention-deficit/hyperactivity disorder (ADHD), zeolite has been suggested as a carrier of L-DOPA, the immediate precursor of dopamine, into the brain of patients, although no clinical studies have been performed so far [238].

## 10. Conclusions

Numerous research shows the positive effects of the naturally occurring zeolite clinoptilolite and its micronized, mechanically activated forms on gut health and microbiota underlined by their detoxifying, anti-inflammatory, immunostimulatory, and antioxidative properties. In recent years a rising number of studies has been relating the pathogenesis and progression of Alzheimer’s disease and Parkinson’s disease to changes in gut function and to gut dysbiosis positing clinoptilolite and its activated forms as a promising new supporting therapy in the treatment of the two diseases. Additional research, however, especially in animal models of the two diseases and possible clinical trials in patients, should shed more light on how effective in AD and PD clinoptilolite and its forms could be.
